# Intraobserver and interobserver agreement of 8 segmental reflexes in healthy dogs

**DOI:** 10.1111/jvim.16999

**Published:** 2024-02-10

**Authors:** Bryan Chiang, Gabriel Garcia, Francesco Leverone, Jorge A. Hernandez, Sheila Carrera‐Justiz

**Affiliations:** ^1^ Small Animal Clinical Sciences College of Veterinary Medicine, University of Florida Gainesville Florida USA; ^2^ Large Animal Clinical Sciences College of Veterinary Medicine, University of Florida Gainesville Florida USA

**Keywords:** canine, neurolocalization, neurological examination, reflexes

## Abstract

**Background:**

No available literature supports the claim that the patellar and withdrawal (flexor) reflexes are the only reliable segmental reflexes in dogs.

**Objective:**

Measure intra‐ and interobserver agreement of 8 segmental reflexes in dogs without clinical evidence of orthopedic or neurologic disease.

**Animals:**

One‐hundred and one client‐ or staff‐owned dogs between 1 and 10 years of age with no clinical evidence of orthopedic disease, myelopathy, or neuromuscular disease.

**Methods:**

Descriptive study. The intraobserver proportion of agreement (%) of responses to selected segmental reflexes in right versus left limbs by 3 observers was calculated and reported. The interobserver agreement of 2 observers of responses to selected reflexes was estimated by calculating proportions of agreement, kappa values, and 95% confidence intervals. A segmental reflex with an acceptable agreement was defined as that with a proportion of agreement ≥90% and a Kappa value ≥0.61 in both limbs.

**Results:**

The intraobserver proportion of agreement for all 3 observers was high (≥95%) for the extensor carpi radialis, withdrawal, patellar, and cranial tibial reflexes. Between observers 1 and 3 and observers 2 and 3, the interobserver proportion of agreement was high (≥ 92%) for the extensor carpi radialis (*κ* 0.66, not determined [ND]), withdrawal (both limbs, *κ* ND), patellar (*κ* ND), and cranial tibial reflexes (*κ* ND).

**Conclusions and Clinical Importance:**

The extensor carpi radialis, withdrawal, patellar, and cranial tibial reflexes had a higher proportion of agreement and kappa values between 2 observers.

## 1 INTRODUCTION

Thorough neurologic evaluation involves the assessment of segmental reflexes,^1^, ^2^, ^3^, ^4^, ^5^ assisting the clinician in properly localizing a lesion within the nervous system. Most limb reflexes are myotatic reflexes, with monosynaptic pathways. The patellar, gastrocnemius, cranial tibial, triceps, biceps, and extensor carpi radialis reflexes traditionally have been considered myotatic reflexes. The withdrawal reflex is not considered to be a myotatic reflex because it requires action of motor neurons from several spinal cord segments.

It is widely accepted that the patellar and withdrawal reflexes are the most reliable.^1^, ^2^, ^3^, ^4^, ^5^ Two studies have evaluated the interobserver agreement of segmental reflexes in dogs. Both studies found the level of experience of the observer has an influence on the interobserver agreement in the biceps reflex, thus that reflex was assessed by observers with the highest level of expertise (ie, neurologists).^6^, ^7^ The second study identified hair coat length as a factor affecting the interpretation of the biceps reflex, as well as level of expertise influencing interobserver agreement for the patellar reflex.^7^ The classification of the cranial tibial and extensor carpi radialis reflexes as myotatic reflexes also recently has been challenged.^8^, ^9^ An unpublished report indicated that the extensor carpi radialis and cranial tibial reflexes were still present in dog cadavers after transection of the radial and sciatic nerves.^8^ This led to a study that found those reflexes persisted after a local block, indicating that the motor response observed for those reflexes may not be dependent solely on the reflex arc, but also an idiomuscular response.^9^

Our objective was to measure the intra‐ and interobserver agreement of 8 segmental reflexes in the limbs of dogs without clinical evidence of orthopedic or neurologic disease by 3 veterinary clinicians. We hypothesized that the patellar and withdrawal reflexes in the thoracic and pelvic limbs would show acceptable agreement and reliable results in clinically normal dogs.

## 2 MATERIALS AND METHODS

The study was performed in accordance with the guidelines of the Institutional Animal Care and Use Committee (IACUC) and the Veterinary Hospital Research Review Committee (VHRRC) of the University of Florida.

### 2.1 Study animals

A convenience sample of 101 client‐ or staff‐owned dogs that were presented to the neurology service at the University of Florida Small Animal Hospital were sequentially included in the study from September 2021 to August 2022. Dogs of any breed, sex, or weight and between 1 and 10 years of age were included. Puppies (<1 year of age), geriatric dogs (>10 years of age), and dogs with clinical evidence of orthopedic disease that inhibited normal range of motion of the limbs, those with myelopathy and neuromuscular diseases, or with suspicion of such diseases, were not included. Any dog that could not tolerate having all of its segmental reflexes tested in lateral recumbency because of temperament also was excluded from the study.

### 2.2 Study design

All study dogs underwent a general physical examination and full neurological examination by a neurology resident (observer 3, BC). The intraobserver proportion of agreement was calculated by measuring the agreement of segmental reflexes of the right limb versus the left limb by 2 observers (the resident and 1 of 2 board‐certified neurologists). The examinations were conducted independently 1 time by each observer. Observer 1 (SCJ) evaluated 65 dogs, observer 2 (GG) evaluated 36 dogs, and observer 3 evaluated 101 dogs. Once the examinations were performed, results were recorded for later analysis.

The interobserver proportion of agreement was calculated by measuring and comparing the proportion of agreement of segmental reflexes between observers 1 and 3, and observers 2 and 3. The segmental reflexes were independently tested 1 time by the observers.

### 2.3 Examination of segmental reflexes

Eight segmental reflexes (4 in the thoracic limb and 4 in the pelvic limb) were tested in every dog on both sides. All of the segmental reflexes were tested on the nondependent limb with the dog in lateral recumbency. The segmental reflexes tested in the thoracic limb were the biceps reflex, triceps reflex, extensor carpi radialis reflex, and the withdrawal reflex. The segmental reflexes tested in the pelvic limb were the patellar reflex, cranial tibial reflex, gastrocnemius reflex, and the withdrawal reflex. All of the reflex responses were classified as positive or negative. The description of how the stimulus is applied and the definition of a positive versus a negative response for each segmental reflex are described as follows (Video S1 available):

*Biceps reflex*: The antebrachium is supported with the limb externally rotated. A finger is placed directly over the tendon of insertion of the biceps brachii and a stimulus is provided by striking the dorsal aspect of the finger overlying the tendon with a pleximeter. A positive response is defined by a contraction of the biceps brachii muscle, or a sudden flexion of the cubital (elbow) joint. A negative response is defined by the absence of contraction of the biceps brachii muscle and absence of flexion of the cubital joint.
*Triceps reflex*: The elbow is flexed to at least 90° and externally rotated by approximately 45°. The tendon of insertion of the triceps brachii is struck with a pleximeter just above its attachment to the olecranon. A positive response is defined by a contraction of the triceps brachii muscle or a sudden extension of the cubital joint. A negative response is defined by the absence of contraction of the triceps brachii muscle and absence of extension of the cubital joint.
*Extensor carpi radialis reflex*: The antebrachium is rested in the nontesting hand of the observer. The location of the muscle belly of the extensor carpi radialis muscle then is estimated by drawing an imaginary line from the lateral epicondyle of the humerus to the mid‐dorsal aspect of the carpus and divided into thirds. The muscle belly of the extensor carpi radialis muscle is located at the most proximal third of the line. The muscle belly is then struck by a pleximeter. A positive response is defined by a sudden extension of the carpal joint. A negative response is defined by the absence of extension of the carpal joint.
*Withdrawal (flexor) reflex in the thoracic limb*: The limb is held in extension and a noxious stimulus is applied to the third or fourth digit by pinching the bone of the digits or the interdigital skin. A positive response is defined by the presence of flexion of the carpus, elbow, and shoulder. A negative response is defined by an absence of flexion of the carpus, elbow, and shoulder.
*Patellar reflex*: The patella is palpated to ensure it is not luxated. If luxated, the patella should be replaced in the patellar groove of the femur. The limb is held with the stifle flexed and the patellar tendon is identified and struck with a pleximeter. A positive response is defined by the presence of a sudden extension of the stifle. A negative response is defined by the absence of extension of the stifle.
*Cranial tibial reflex*: The limb is held resting in the nontesting hand of the observer. The muscle belly of the cranial tibial muscle is identified in the proximal region of the craniolateral aspect of the tibia. The largest area of the muscle belly is struck with a pleximeter. A positive response is defined by a sudden flexion of the tarsus. A negative response is defined by the absence of flexion at the tarsus.
*Gastrocnemius reflex*: The metatarsus is held with the stifle in extension and the hock is flexed and externally rotated to load the calcaneal tendon. The common calcaneal tendon just proximal to its insertion at the calcaneus is struck with a pleximeter. A positive response is defined by a sudden extension of the tarsus or contraction of the gastrocnemius muscle or the caudal thigh muscles. A negative response is defined by the absence of a contraction of the gastrocnemius and caudal thigh muscles and absence of extension of the hock.
*Withdrawal (flexor) reflex of the pelvic limb*: This reflex is tested in similar fashion as the withdrawal reflex in the thoracic limb. A positive response is defined by the presence of flexion of the hock, stifle, and hip. A negative response is defined by the absence of flexion of the hock, stifle, and hip.

### 2.4 Statistical analysis

For each observer, the intraobserver agreement of dog responses to selected segmental reflexes in right versus left limbs was calculated by dividing the sum of the total number of dogs with negative responses in left and right limbs and the total number of dogs with positive responses in both limbs by the total number of dogs tested. Interobserver agreement was measured by calculating the observed proportion of agreement. In this study, kappa values (*k*) and 95% confidence intervals (CI) were calculated and reported as measures of reliability. Kappa values were interpreted as follows: excellent agreement (0.93‐1.00); very good agreement (0.81‐0.92); good agreement (0.61‐0.80); fair agreement (0.41‐0.60); slight agreement (0.21‐0.40); poor agreement (0.01‐0.20); no agreement (≤0.00).^10^, ^11^ Kappa analysis was conducted using commercial software (MedCalc Statistical Software version 20.111, MedCalc Software Ltd, Ostend, Belgium; https://www.medcalc.org; 2022). For selected comparisons with perfect (100%) or near perfect (98%) observed agreement, kappa was not determined (ND) because the frequency of dogs in selected categories was zero. A segmental reflex was defined as reliable if it had an observed proportion of agreement ≥90% and a kappa value ≥0.61 in both right and left limbs.

## 3 RESULTS

### 3.1 Dogs

One‐hundred and ten dogs (3 intact males, 51 neutered males, 4 intact females, and 52 spayed females) were recruited to participate in the study. Eight dogs (6 neutered males and 2 spayed females) were excluded from the study because their temperaments did not allow for testing of the reflexes in lateral recumbency. One additional dog (6.5‐year‐old, intact male Golden retriever) was excluded because the dog developed neck pain the day before examination. A total of 101 dogs met the inclusion criteria. Breeds represented included mixed breed (n = 46), Golden retriever (n = 6), Labrador retriever (n = 6), American Staffordshire terrier (n = 4), Australian shepherd (n = 3), French bulldog (n = 3), Boston terrier (n = 2), bull terrier (n = 2), Cavalier King Charles spaniel (n = 2), Shetland sheepdog (n = 2), Weimaraner (n = 2), and 1 of each of the following breeds: American bulldog, Anatolian shepherd, Australian cattle dog, Basset hound, Belgian Malinois, Boxer, Coonhound, Dachshund, English setter, Flat‐coated retriever, German shepherd, German shorthaired pointer, Giant Schnauzer, Great Dane, Greyhound, Jack Russell terrier, Miniature Poodle, Neapolitan mastiff, Pembroke Welsh corgi, Pug, Rottweiler, Whippet, and Yorkshire terrier. There were 54 females (4 intact, 50 spayed) and 47 males (2 intact, 45 neutered). The median age of dogs in the study was 4.25 years (range, 1‐9.75 years). Eighty‐seven dogs had normal neurologic examinations. Fourteen dogs were neurolocalized to the forebrain, and 1 dog neurolocalized to the cerebellum. Nine of the 14 forebrain‐localized dogs had been diagnosed with idiopathic epilepsy or a behavioral disorder. The other 5 dogs were diagnosed with meningoencephalitis of unknown origin (4) or a meningioma (1). The dog with a cerebellar localization had a normal neurological examination and brain magnetic resonance imaging (MRI), and had episodes of cerebellar ataxia.

### 3.2 Intra‐ and interobserver agreement

The intraobserver agreement for all 3 observers was high (≥95%) for the extensor carpi radialis reflex, withdrawal reflex (thoracic and pelvic limbs), patellar reflex, and cranial tibial reflex (Tables 1, 2, 3). Similarly, between observers 1 and 3 and observers 2 and 3, the interobserver agreement was high (≥ 92%) for the extensor carpi radialis reflex, withdrawal reflex (thoracic and pelvic limbs), patellar reflex, and cranial tibial reflex (Tables 4, 5, 6, 7).

**TABLE 1:** Intraobserver agreement results of selected reflexes in study dogs by Observer 1.

	Right negative	Right positive	Total	Observed agreement %
Biceps reflex				
Left negative	5	1	6	94
Left positive	3	56	59	
Total	8	57	65	
Triceps reflex				
Left negative	47	2	49	94
Left positive	2	14	16	
Total	49	16	65	
Extensor carpi radialis reflex				
Left negative	6	2	8	95
Left positive	1	56	57	
Total	7	58	65	
Withdrawal reflex (forelimbs)				
Left negative	0	0	0	100
Left positive	0	65	65	
Total	0	65	65	
Patellar reflex				
Left negative	0	0	0	100
Left positive	0	65	65	
Total	0	65	65	
Cranial tibial reflex				
Left negative	0	0	0	98
Left positive	1	64	65	
Total	1	64	65	
Gastrocnemius reflex				
Left negative	32	2	34	95
Left positive	1	30	31	
Total	33	32	65	
Withdrawal reflex (hind limb)				
Left negative	0	0	0	100
Left positive	0	65	65	
Total	0	65	65	

**TABLE 2:** Intraobserver agreement results of selected reflexes in study dogs by Observer 2.

	Right negative	Right positive	Total	Observed agreement %
Biceps reflex				
Left negative	1	2	3	94
Left positive	0	33	33	
Total	1	35	36	
Triceps reflex				
Left negative	5	5	10	67
Left positive	7	19	26	
Total	12	24	36	
Extensor carpi radialis reflex				
Left negative	0	0	0	100
Left positive	0	36	36	
Total	0	36	36	
Withdrawal reflex (forelimbs)				
Left negative	0	0	0	100
Left positive	0	36	36	
Total	0	36	36	
Patellar reflex				
Left negative	0	0	0	100
Left positive	0	36	36	
Total	0	36	36	
Cranial tibial reflex				
Left negative	0	0	0	100
Left positive	0	36	36	
Total	0	36	36	
Gastrocnemius reflex				
Left negative	2	1	3	86
Left positive	4	29	33	
Total	6	30	36	
Withdrawal reflex (hind limb)				
Left negative	0	0	0	100
Left positive	0	36	36	
Total	0	36	36	

**TABLE 3:** Intraobserver agreement results of selected reflexes in study dogs by Observer 3.

	Right negative	Right positive	Total	Observed agreement %
Biceps reflex				
Left negative	10	7	17	88
Left positive	5	79	84	
Total	15	86	101	
Triceps reflex				
Left negative	42	12	54	69
Left positive	19	28	47	
Total	61	40	101	
Extensor carpi radialis reflex				
Left negative	5	4	9	96
Left positive	0	92	92	
Total	5	96	101	
Withdrawal reflex (forelimbs)				
Left negative	0	0	0	100
Left positive	0	101	101	
Total	0	101	101	
Patellar reflex				
Left negative	0	1	1	99
Left positive	0	100	100	
Total	0	101	101	
Cranial tibial reflex				
Left negative	0	0	0	100
Left positive	0	101	101	
Total	0	101	101	
Gastrocnemius reflex				
Left negative	25	11	36	71
Left positive	18	47	65	
Total	43	58	101	
Withdrawal reflex (hind limb)				
Left negative	0	0	0	100
Left positive	0	101	101	
Total	0	101	101	

**TABLE 4:** Interobserver agreement and kappa results of selected reflexes in study dogs between Observer 1 and 3: left limb only.

Biceps reflex				
	Observer 1			
Observer 3	Negative	Positive	Total	Observed agreement 92%
Negative	6	5	11	
Positive	0	54	54	Kappa 0.66 (0.40, 0.93)
Total	6	59	65	

Triceps reflex				
	Observer 1			
Observer 3	Negative	Positive	Total	Observed agreement 70%
Negative	34	4	38	
Positive	15	12	27	Kappa 0.36 (0.14, 0.58)
Total	49	16	65	

Extensor carpi radialis reflex				
	Observer 1			
Observer 3	Negative	Positive	Total	Observed agreement 92%
Negative	6	3	9	
Positive	2	54	56	Kappa 0.66 (0.38, 0.93)
Total	8	57	65	

Withdrawal reflex (forelimbs)				
	Observer 1			
Observer 3	Negative	Positive	Total	Observed agreement 100%
Negative	0	0	0	
Positive	0	65	65	Kappa ND
Total	0	65	65	

Patellar reflex				
	Observer 1			
Observer 3	Negative	Positive	Total	Observed agreement 98%
Negative	0	1	1	
Positive	0	64	64	Kappa ND
Total	0	65	65	

Cranial tibial reflex				
	Observer 1			
Observer 3	Negative	Positive	Total	Observed agreement 100%
Negative	0	0	0	
Positive	0	65	65	Kappa ND
Total	0	65	65	

Gastrocnemius reflex				
	Observer 1			
Observer 3	Negative	Positive	Total	Observed agreement 72%
Negative	22	6	28	
Positive	12	25	37	Kappa 0.44 (0.23, 0.66)
Total	34	31	65	

Withdrawal reflex (hind limb)				
	Observer 1			
Observer 3	Negative	Positive	Total	Observed agreement 100%
Negative	0	0	0	
Positive	0	65	65	Kappa ND
Total	0	65	65	

Abbreviation: ND, not determined because the frequency of dogs in selected categories was zero.

**TABLE 5:** Interobserver agreement and kappa results of selected reflexes in study dogs between Observer 1 and 3: right limb only.

Biceps reflex				
	Observer 1			
Observer 3	Negative	Positive	Total	Observed agreement 86%
Negative	5	6	11	
Positive	3	51	54	Kappa 0.44 (0.14, 0.74)
Total	8	57	65	

Triceps reflex				
	Observer 1			
Observer 3	Negative	Positive	Total	Observed agreement 75%
Negative	38	5	43	
Positive	11	11	22	Kappa 0.41 (0.17, 0.64)
Total	49	16	65	

Extensor carpi radialis reflex				
	Observer 1			
Observer 3	Negative	Positive	Total	Observed agreement 94%
Negative	4	1	5	
Positive	3	57	60	Kappa 0.63 (0.30, 0.96)
Total	7	58	65	

Withdrawal reflex (forelimbs)				
	Observer 1			
Observer 3	Negative	Positive	Total	Observed agreement 100%
Negative	0	0	0	
Positive	0	65	65	Kappa ND
Total	0	65	65	

Patellar reflex				
	Observer 1			
Observer 3	Negative	Positive	Total	Observed agreement 100%
Negative	0	0	0	
Positive	0	65	65	Kappa ND
Total	0	65	65	

Cranial tibial reflex				
	Observer 1			
Observer 3	Negative	Positive	Total	Observed agreement 98%
Negative	0	0	0	
Positive	1	64	65	Kappa ND
Total	1	64	65	

Gastrocnemius reflex				
	Observer 1			
Observer 3	Negative	Positive	Total	Observed agreement 69%
Negative	22	9	31	
Positive	11	23	34	Kappa 0.38 (0.16, 0.60)
Total	33	32	65	

Withdrawal reflex (hind limb)				
	Observer 1			
Observer 3	Negative	Positive	Total	Observed agreement 100%
Negative	0	0	0	
Positive	0	65	65	Kappa ND
Total	0	65	65	

**TABLE 6:** Interobserver agreement and kappa results of selected reflexes in study dogs between Observer 2 and 3: left limb only.

Biceps reflex				
	Observer 2			
Observer 3	Negative	Positive	Total	Observed agreement 92%
Negative	3	3	6	
Positive	0	30	30	Kappa 0.62 (0.24, 1.0)
Total	3	33	36	

Triceps reflex				
	Observer 2			
Observer 3	Negative	Positive	Total	Observed agreement 83%
Negative	10	6	16	
Positive	0	20	20	Kappa 0.64 (0.40, 0.88)
Total	10	26	36	

Extensor carpi radialis reflex				
	Observer 2			
Observer 3	Negative	Positive	Total	Observed agreement 100%
Negative	0	0	0	
Positive	0	36	36	Kappa ND
Total	0	36	36	

Withdrawal reflex (forelimbs)				
	Observer 2			
Observer 3	Negative	Positive	Total	Observed agreement 100%
Negative	0	0	0	
Positive	0	36	36	Kappa ND
Total	0	36	36	

Patellar reflex				
	Observer 2			
Observer 3	Negative	Positive	Total	Observed agreement 100%
Negative	0	0	0	
Positive	0	36	36	Kappa ND
Total	0	36	36	

Cranial tibial reflex				
	Observer 2			
Observer 3	Negative	Positive	Total	Observed agreement 100%
Negative	0	0	0	
Positive	0	36	36	Kappa ND
Total	0	36	36	

Gastrocnemius reflex				
	Observer 2			
Observer 3	Negative	Positive	Total	Observed agreement 81%
Negative	2	6	8	
Positive	1	27	28	Kappa 0.27 (0.0, 0.64)
Total	3	33	36	

Withdrawal reflex (hind limb)				
	Observer 2			
Observer 3	Negative	Positive	Total	Observed agreement 100%
Negative	0	0	0	
Positive	0	36	36	Kappa ND
Total	0	36	36	

**TABLE 7:** Interobserver agreement and kappa results of selected reflexes in study dogs between Observer 2 and 3: right limb only.

Biceps reflex				
	Observer 2			
Observer 3	Negative	Positive	Total	Observed agreement 92%
Negative	1	3	4	
Positive	0	32	32	Kappa 0.37 (0.0, 0.90)
Total	1	35	36	

Triceps reflex				
	Observer 2			
Observer 3	Negative	Positive	Total	Observed agreement 72%
Negative	10	8	18	
Positive	2	16	18	Kappa 0.44 (0.16, 0.72)
Total	12	24	36	

Extensor carpi radialis reflex				
	Observer 2			
Observer 3	Negative	Positive	Total	Observed agreement 100%
Negative	0	0	0	
Positive	0	36	36	Kappa ND
Total	0	36	36	

Withdrawal reflex (forelimbs)				
	Observer 2			
Observer 3	Negative	Positive	Total	Observed agreement 100%
Negative	0	0	0	
Positive	0	36	36	Kappa ND
Total	0	36	36	

Patellar reflex				
	Observer 2			
Observer 3	Negative	Positive	Total	Observed agreement 100%
Negative	0	0	0	
Positive	0	36	36	Kappa ND
Total	0	36	36	

Cranial tibial reflex				
	Observer 2			
Observer 3	Negative	Positive	Total	Observed agreement 100%
Negative	0	0	0	
Positive	0	36	36	Kappa ND
Total	0	36	36	

Gastrocnemius reflex				
	Observer 2			
Observer 3	Negative	Positive	Total	Observed agreement 83%
Negative	6	6	12	
Positive	0	24	24	Kappa 0.57 (0.28, 0.85)
Total	6	30	36	

Withdrawal reflex (hind limb)				
	Observer 2			
Observer 3	Negative	Positive	Total	Observed agreement 100%
Negative	0	0	0	
Positive	0	36	36	Kappa ND
Total	0	36	36	

## 4 DISCUSSION

The observation of segmental reflexes still plays an integral role in neurolocalization for the practitioner despite the subjective interpretation of the findings. Our results support the hypothesis that the patellar and withdrawal reflexes would be reliably present in the study population of dogs. Based on the frequency of positive responses and agreement between observers, additional reflexes that were considered reliably present in our study sample include the extensor carpi radialis reflex and the cranial tibial reflex.

The patellar reflex and the withdrawal reflex have been widely accepted as the 2 most reliable reflexes in dogs, in the sense of how frequently they are present in normal dogs as well as how accurate a decreased or increased reflex response is in localizing a lesion.^1^, ^2^, ^3^, ^4^, ^5^ Several studies have examined the accuracy of these reflexes in localizing a lesion. A retrospective study investigated the accuracy of the withdrawal reflex in localizing a cervical intervertebral disc herniation in dogs compared with the MRI findings.^12^ This study concluded that the withdrawal reflex was not reliable in its ability to localize a lesion to the C1‐C5 vs the C6‐T2 segments. A second study assessed the accuracy of neurologic examination findings in localizing the site of thoracolumbar intervertebral disc herniations in dogs.^13^ A decreased withdrawal reflex correctly predicted a lesion in the L4‐S3 region only 37.5% of the time. In that same study evaluating the patellar reflex, a decreased patellar reflex correctly predicted a lesion in the L4‐S3 region only 16.7% of the time whereas an increased patellar reflex was more accurate (87%) in predicting a lesion in the T3‐L3 region. A third study investigated the accuracy of the patellar reflex in localizing a thoracolumbar disc extrusion.^14^ All of the dogs in that study with a decreased patellar reflex had lesions within the T3‐L3 region.

On the other hand, only 2 studies have investigated spinal reflexes in normal dogs. Both of these studies identified that the experience of the examiner can influence the interobserver agreement of the biceps reflex.^10^, ^11^ If the examiner is very experienced, the biceps reflex can be a reliable reflex. This hypothesis was supported by the intraobserver agreements for the biceps reflex in our study. The agreement was higher for the 2 neurologists (observers 1 and 2) compared with a lower agreement for observer 3 who was a neurology resident. In the other study, long‐haired dogs had a 2.6‐fold increased chance of disagreement for the biceps reflex compared with shorthaired dogs, and interobserver agreement was found to be better with higher levels of expertise for the patellar reflex.^7^ Sixteen dogs in our study were breeds with long hair coats (eg, Golden retriever, Cavalier King Charles spaniel, Australian Shepherd, Shetland sheepdog, Flat‐coated retriever, Yorkshire terrier, and English setter).

Even with the inclusion of these dogs, our findings still suggest the extensor carpi radialis reflex, patellar reflex, cranial tibial reflex, withdrawal reflex, and possibly the biceps reflex are reliably present in dogs. Our findings also support the hypothesis that a more experienced observer can influence the observed agreement on the biceps reflex. The intraobserver agreement for the biceps reflex for both of the board‐certified neurologists (observers 1 and 2) was 94% whereas the intraobserver agreement for the biceps reflex for the neurology resident (observer 3) was 88%. Additionally, the interobserver agreement was reliable only for the left limb but not the right which may be explained by the technique used to isolate the tendon of insertion of the biceps brachii and how the animal was positioned in reference to where the observer was in testing the reflex, which could affect how well the reflex could be observed. Video analysis may have helped in the interpretation of subtle responses that could be easily missed during clinical examination.

In our study, the extensor carpi radialis reflex and the cranial tibial reflex were classified as reliable for neurologic examinations in dogs. Compared with the extensor carpi radialis reflex, the cranial tibial reflex is more easily tested. The patellar tendon is easily palpated. The cranial tibialis muscle has a large muscle belly at the cranio‐proximal region of the tibia that is easily palpated. The withdrawal reflex is tested in the form of a noxious stimulus by pinching the toes. The responses generated for these 3 reflexes are not subtle or difficult to observe. The extensor carpi radialis muscle on the other hand is a smaller muscle and difficult to palpate externally. It lies in proximity to the common digital extensor and lateral digital extensor muscles that also serve to extend the carpus. If the examiner mistakenly strikes one of the other carpal extensors, the response is likely still observed as an extension of the carpus.

A previous study comparing the cranial tibial and extensor carpi radialis reflexes in cats concluded that these 2 reflexes are not strictly myotatic reflexes and may represent an idiomuscular response.^9^ Idiomuscular responses were first described in 1858^15^ and are a result of direct activation of the sarcolemma after direct percussion of the muscle.^16^ This method is the way in which both the extensor carpi radialis and cranial tibial reflexes are tested in dogs and cats. Idiomuscular responses are not commonly used in human medicine because of their uncertain mechanism and clinical relevance.^15^ However, differences in the character of the response in testing the cranial tibial reflex in which it demonstrated more of a brisk dorsiflexion of the ankle in patients with lower motor neuron signs versus an inversion of the ankle in patients with upper motor neuron lesions seemed helpful in the assessment of patients with lower limb weakness.^15^ The correlation to veterinary medicine is likely not helpful because these subtle differences in response would be difficult to appreciate. We elected to include these reflexes in our study because they traditionally are taught as part of a complete neurological examination.^2^, ^3^, ^4^, ^5^, ^17^ Although idiomuscular responses may be easy to perform, the presence or absence of these responses may not correlate with neurolocalization of the lesion.

Our study had several limitations. First, the number of animals examined by each of the observers was not equal. This factor could have skewed the results and the observed agreements. Each observer made his or her own interpretations of the reflexes examined, which could introduce bias. Video‐taped responses by the most experienced observer with subsequent blinded analysis of the responses by observers of various levels of experience could have removed this bias. Second, some animals included in our study had abnormalities on their neurologic examinations. Advanced imaging was not performed to exclude the possibility of spinal lesions in all of the animals included. Therefore, some of the dogs may have had spinal lesions. However, all of these dogs were neuro‐localized to the brain and were suspected not to have any concurrent myelopathies. Third, although the calculated interobserver agreement and kappa statistic value were high for several investigated reflexes, it is important to examine all calculated outcomes for agreement. For example, for the biceps reflex (observer 1 vs 3, left limb), the observed agreement was 60/65 or 92%, the calculated kappa value was 0.66, and the 95% CI was 0.44 to 0.90. The width of the CI is strongly dependent on sample size.^18^ The calculated kappa value (0.66) represents the maximum point estimate in the distribution of the CI, and the lower and upper limits are very unlikely to be values in that distribution. If the true kappa value were 0.66, a sample size 2 times higher (ie, 130 dogs) than that used for the biceps reflex would have resulted in a narrower 95% CI (ie, 0.48‐0.65), decreasing the magnitude of uncertainty associated with the calculated agreement for the biceps reflex. In our study, 2 crucial investigated reflexes were the patellar and withdrawal reflexes. In both reflexes, the calculated observed agreements were 64/65 or 98% and 65/65 or 100% but kappa values were not calculated because the frequency of dogs in selected categories was 1 or zero. Finally, the external validity in our study was inherently low because the study sample was limited to a convenience sample of up to 101 client‐ or staff‐owned dogs that were presented to a single university hospital for veterinary care. Thus, study results apply to the study sample of investigated dogs and cannot be extrapolated to other dog populations.

## 5 CONCLUSION

The patellar and the withdrawal reflexes in both the thoracic and pelvic limbs, as well as the extensor carpi radialis and the cranial tibial reflexes, were classified as reliable in a study sample of 101 dogs. Our findings support inclusion of the patellar and withdrawal reflexes in all neurological examinations.

## CONFLICT OF INTEREST DECLARATION

Authors declare no conflict of interest.

## OFF‐LABEL ANTIMICROBIAL DECLARATION

Authors declare no off‐label use of antimicrobials.

## INSTITUTIONAL ANIMAL CARE AND USE COMMITTEE (IACUC) OR OTHER APPROVAL DECLARATION

Approved by the College of Veterinary Medicine Hospital Research Review Committee and the University of Florida IACUC, study number 202111318.

## HUMAN ETHICS APPROVAL DECLARATION

Authors declare human ethics approval was not needed for this study.

## Supporting information


**Table S1.** Demographic information, neurolocalization, and history of 101 dogs.

**Video S1.** This video captures positioning used for all reflexes evaluated as well as positive responses.
